# Divergent Effects of Calcium Channel Modulators on H-Reflex Excitability in Fatigued Rat Muscle

**DOI:** 10.3390/ijms262110749

**Published:** 2025-11-05

**Authors:** Andriy Maznychenko, Tetiana I. Abramovych, Nataliya V. Bulgakova, Vasyl Melenko, Yuliia A. Levchuk, Tatyana Shevchuk, Inna Sokolowska, Alexander I. Kostyukov

**Affiliations:** 1 Department of Physical Education, Gdansk University of Physical Education and Sport, Kazimierza Gorskiego Str. 1, 80-336 Gdansk, Poland; 2 Department of Movement Physiology, Bogomoletz Institute of Physiology NAS of Ukraine, Bogomoletz Str. 4, 01024 Kyiv, Ukraine; 3 Department of Spinal Surgery, State Institution “Institute of Traumatology and Orthopedics”, National Academy of Medical Science of Ukraine, Bulvarno-Kudriavska Str. 27, 01054 Kyiv, Ukraine; 4 Surgery Department, Municipal Nonprofit Enterprise “Kyiv City Clinical Hospital #4”, Solomyanska Str. 17, 03110 Kyiv, Ukraine; 5 Anatomy Department, Lesia Ukrainka Volyn National University, Voli Ave. 13, 43025 Lutsk, Ukraine

**Keywords:** skeletal muscle fatigue, Ca^2+^ channel modulators, Amiloride, Nifedipine, (−)-Bay K8644, H-reflex amplitude, electrical stimulation, rat

## Abstract

Calcium (Ca^2+^) release from the sarcoplasmic reticulum is central to excitation–contraction coupling and plays a critical role in the development of skeletal muscle fatigue. Altered Ca^2+^ dynamics may affect not only contractile function but also neuromuscular excitability. This study examined the effects of pharmacological modulation of Ca^2+^ channels on fatigue development and spinal reflex activity in rats. Using the Hoffmann reflex (H-reflex) as an indicator of motoneuron excitability, we evaluated the effects of Ca^2+^ channel blockers (Amiloride, Nifedipine) and an activator ((−)-Bay K8644) on the reflex responses of the plantar muscle before and after fatigue induction. The ratio of the maximum H-reflex to maximum M-wave (H_max_/M_max_) was used to assess alterations in spinal excitability. Compared with the control, both Amiloride and Nifedipine markedly reduce the H_max_/M_max_ ratio (77% and 60%, respectively), whereas (−)-Bay K8644 elicited a robust 129% increase. These findings demonstrate that pharmacological modulation of Ca^2+^ channels has distinct and divergent effects on spinal excitability during fatigue. These results highlight the close interaction between intramuscular Ca^2+^ regulation and reflex pathways and suggest potential strategies for enhancing muscle performance through targeted Ca^2+^ channel modulation.

## 1. Introduction

Fatigue is a complex physiological phenomenon that arises from the intricate interplay of central and peripheral mechanisms that act at different levels of the neuromuscular system [1,2]. At the peripheral level, the regulation of calcium (Ca^2+^) within skeletal muscle fibers is recognized as a fundamental determinant of contractile performance, since Ca^2+^ released from the sarcoplasmic reticulum (SR) binds to troponin C, initiates cross-bridge cycling, and ultimately drives muscle contraction [3]. Disturbances in Ca^2+^ homeostasis, whether through impaired release, reuptake, or buffering, contribute to the decline in force-generating capacity that is characteristic of muscular fatigue [4,5]. However, fatigue is not exclusively a peripheral event; it also reflects changes in neural excitability, synaptic transmission, and motor unit recruitment, processes that are regulated at the spinal level [6]. The initiation of muscle contraction involves the release of acetylcholine, leading to sarcolemma depolarization and subsequent rapid changes in transmembrane potential, which are orchestrated by the dihydropyridine receptor Ca^2+^ channel (DHPR) and the ryanodine receptor RyR1, resulting in the rapid release of Ca^2+^ into the cytoplasm [7,8]. This transient increase in the cytosolic Ca^2+^ concentration initiates force production. As fatigue develops, multiple mechanisms contribute to impaired Ca^2+^ handling. These include reduced sensitivity of the contractile apparatus to Ca^2+^, depletion of SR Ca^2+^ stores, oxidative modifications of RyRs, and impaired SR Ca^2+^ ATPase activity [4]. Collectively, these alterations diminish the ability of the muscle to sustain force. The link between intramuscular Ca^2+^ regulation and spinal excitability can be understood within the broader framework of sensorimotor integration. Skeletal muscle is not only an effector organ but also a key source of afferent feedback to the spinal cord [9]. Group Ia afferents that arise from muscle spindles and group Ib afferents from Golgi tendon organs continuously inform the central nervous system about muscle length, velocity, and tension. Fatigue-induced alterations in muscle contractile properties can modify this sensory feedback [10]. For example, changes in Ca^2+^ handling influence muscle fiber stiffness, twitch characteristics, and the time-course of relaxation, all of which affect the discharge patterns of muscle spindle afferents [11]. Moreover, metabolites that accumulate during fatigue, such as lactate, inorganic phosphate, and H^+^, can alter the excitability of group III and IV muscle afferents, further modulating spinal reflex circuits [12]. Thus, pharmacological manipulation of Ca^2+^ flux within the muscle may indirectly influence spinal excitability by altering the sensory input that drives reflex responses [13]. Such modulation of Ca^2+^ channels provides a valuable approach to dissect the relationship between excitation–contraction coupling and neuromuscular excitability. Nifedipine, a classic L-type Ca^2+^ channel blocker, reduces Ca^2+^ influx through voltage-gated channels and limits sarcoplasmic Ca^2+^ release [14]. Amiloride, primarily an inhibitor of Na^+^/Ca^2+^ exchange, also interferes with Ca^2+^ handling at the muscle membrane [15]. In contrast, (−)-Bay K8644 acts as an L-type Ca^2+^ channel agonist, prolonging channel opening and enhancing Ca^2+^ entry [16]. However, their implications for skeletal muscle fatigue and neuromuscular reflex activity remain poorly defined. Employing these agents in a fatigue model enables the controlled manipulation of intramuscular Ca^2+^ dynamics and the evaluation of their consequences for spinal reflex activity.

The Hoffmann reflex (H-reflex), a well-established electrophysiological tool, provides an index of spinal motoneuron excitability and has been widely used to evaluate adaptations in central drive and reflex regulation under conditions of fatigue, training, or pharmacological intervention [13,17,18]. Modulation of the H-reflex amplitude, coupled with a specific stimulus, is frequently utilized to evaluate postsynaptic events or alterations in the extent of presynaptic inhibition affecting Ia afferent terminals. This stems from the interaction between motor neuron excitability and the ongoing presynaptic inhibition of Ia fibers responsible for conveying the test afferent volley, thereby impacting the amplitude of the test reflex [19]. The H-reflex technique, which is used to assess the excitability of α-motoneurons [20], provides valuable insights into muscle fatigue evaluation. The ratio of the maximum H-reflex amplitude to the maximum M-wave amplitude (H_max_/M_max_) reflects the percentage of activated α-motoneurons during electrical stimulation. H_max_ provides information about the maximum number of recruited motor units, whereas M_max_ provides data on the absolute number of motor units [21]. These parameters offer a means to assess the nervous system’s response to various stimuli. Despite the growing body of evidence linking Ca^2+^ dynamics to muscle performance, the potential influence of pharmacological modulation of Ca^2+^ channels on spinal excitability, as indexed by the H-reflex, remains largely unexplored. Only a few investigations (e.g., [13]) have combined the effects of Ca^2+^ channel modulators and H-reflexometry to explore how manipulation of peripheral Ca^2+^ handling might be reflected in spinal reflex pathways. Understanding how Ca^2+^ channel modulators influence both fatigue development and spinal excitability could provide novel insights into therapeutic strategies aimed at prolonging muscle performance or modulating reflex activity in clinical and athletic contexts. By analyzing the H_max_/M_max_ ratio, we sought to determine whether pharmacological modulation of Ca^2+^ channels alters spinal excitability in fatigued muscle.

The aim of this study was to determine whether pharmacological modulation of calcium channels alters spinal excitability during the development of muscle fatigue, as assessed by the H-reflex parameters. Specifically, we examined the effects of two calcium channel blockers (Amiloride and Nifedipine) and one activator ((−)-Bay K8644) on the H_max_/M_max_ ratio in fatigued rat skeletal muscle. We hypothesized that the blockers would reduce H-reflex amplitude, reflecting decreased afferent input or motoneuron responsiveness, whereas the activator would preserve or enhance reflex excitability. This approach allowed us to address the central research question: how do distinct classes of calcium channel modulators influence spinal reflex activity during fatigue?

## 2. Results

**Figure 1:**
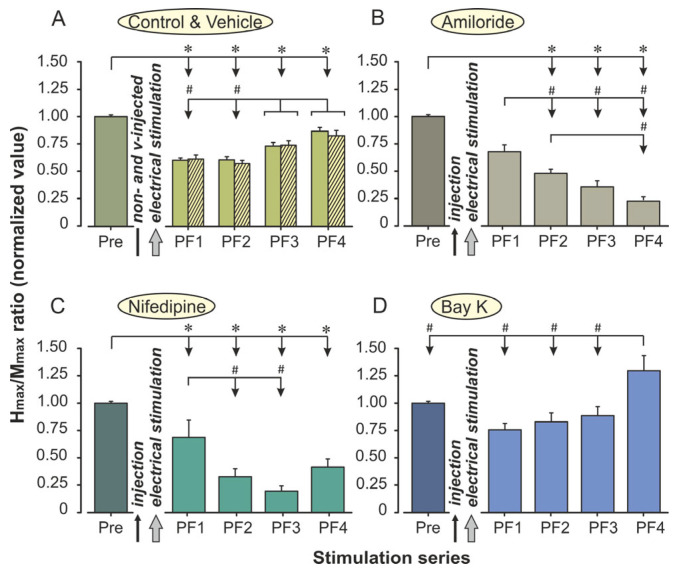
Effects of calcium channel modulators on H-reflex excitability during fatigue development in rat *m. plantaris*. (**A**) Non-injected control group and vehicle-treated animals. (**B**) Amlodipine (1 mg/kg, i.p.). (**C**) Nifedipine (5 mg/kg, i.p.). (**D**) (−)-Bay K 8644 (0.5 mg/kg, i.p.). Data represent normalized H_max_/M_max_ ratios (mean ± SEM) recorded before fatigue induction (Pre) and during four postfatigue periods (PF1–PF4). Asterisks indicate significant differences between the Pre and PF time points (*p* < 0.05); the # symbol denotes significant differences among PF1–PF4 values (*p* < 0.05). For all groups, *n* = 8.

Electrical stimulation induced marked, time-dependent changes in the H_max_/M_max_ ratio, which differed across experimental groups. A two-way repeated measures ANOVA was performed with the Time (one prefatigue [Pre] and four postfatigue registrations [PF1–PF4]) as the within-subjects factor and the Group (Control, Amiloride, Nifedipine, (−)-Bay K8644) as the between-subjects factor. This analysis assessed whether pharmacological modulation of calcium channels influenced the magnitude and recovery dynamics of the H-reflex following a standardized fatiguing protocol. No significant differences were observed between animals in the control and vehicle-injected groups; therefore, results are presented using the control group values. Group-wise comparisons of the H_max_/M_max_ ratios across time points are shown in Figure 1A.

Mauchly’s test of sphericity showed that the assumption of sphericity was violated (*W* = 0.368, *p* = 0.0018). Consequently, Greenhouse–Geisser correction (ε = 0.698) was applied to all within-subjects effects involving the Time factor. The use of the corrected degrees of freedom ensured an accurate estimation of significance levels given the unequal variances among repeated measures.

Analysis revealed a highly significant main effect of Time, *F*(2.79, 78.21) = 35.34, *p* < 0.0001, η^2^_p_ = 0.56, 95% CI [0.50, 0.60], demonstrating that repeated stimulation produced consistent modifications in reflex amplitude throughout the protocol. Across all groups, the H_max_/M_max_ ratio initially decreased after drug administration (PF1) and further declined during the fatiguing stimulation periods (PF2 and PF3). In most animals, this suppression was followed by partial recovery during the final recording (PF4). On average, reflex amplitude dropped by approximately 35–40% from baseline at the peak of fatigue and recovered to about 80–85% of the initial value by PF4, indicating a substantial though incomplete restoration of motoneuron excitability.

A significant main effect of the Group was also found, *F*(3, 28) = 27.05, *p* < 0.0001, η^2^_p_ = 0.74, 95% CI [0.67, 0.77], showing that the different pharmacological treatments produced distinct overall levels of H-reflex excitability. When values were averaged across all time points, the highest mean amplitudes were observed in the (−)-Bay K8644 group, followed by the Control group, while the Amiloride and Nifedipine groups exhibited markedly lower responses. These findings confirm that modulation of calcium influx exerts a strong influence on the general excitability of the spinal reflex pathway.

Importantly, a significant Group × Time interaction was detected, *F*(8.38, 78.21) = 10.41, *p* < 0.0001, η^2^_p_ = 0.53, 95% CI [0.47, 0.57], indicating that the temporal profile of H-reflex modulation differed substantially among groups. In the Control group, stimulation induced a pronounced but transient reduction in reflex amplitude at PF1 and PF2, followed by gradual recovery toward baseline during the final recording period. In contrast, both Amiloride- and Nifedipine-treated animals showed a more persistent and deeper suppression of the reflex, which remained significantly below baseline throughout the entire postfatigue phase (*p* < 0.01). The recovery observed in these groups was slow and incomplete, suggesting prolonged depression of motoneuron responsiveness.

In sharp contrast, the (−)-Bay K8644 treatment resulted in a distinctly different response pattern. Reflex amplitude remained relatively stable after the injection phase and declined only slightly during fatiguing stimulation. By the recovery stage (PF4), the H-reflex had not only returned to baseline but exceeded prestimulation levels in several animals. Post hoc comparisons confirmed that the H_max_/M_max_ ratios in the (−)-Bay K8644 group were significantly higher than in the Amiloride and Nifedipine groups at PF3 and PF4 (*p* < 0.01) and also higher than in controls at PF4 (*p* = 0.019). This pattern suggests a facilitating influence of calcium-channel activation on postfatigue recovery mechanisms.

To further explore within-group dynamics, one-way repeated measures ANOVAs were conducted separately for each condition. In the Control group, a significant effect of Time was confirmed (*F*(2.60, 18.23) = 83.27, *p* < 0.0001). Immediately after electrical stimulation, the H_max_/M_max_ ratio decreased by approximately 40% (in comparison with Pre) and remained strongly suppressed during the second postfatigue period (PF2 ≈ 61%). Partial recovery was observed thereafter, with excitability increasing to 74% at PF3 and 88% at PF4 (Figure 1A). Despite this progressive improvement, the reflex remained below prefatigue levels (*p* < 0.001). Bonferroni comparisons confirmed that each consecutive recovery phase differed significantly from the preceding one (*p* < 0.001), demonstrating a gradual but incomplete restoration of motoneuron responsiveness.

In animals treated with Amiloride, the H-reflex excitability showed a strong and progressive suppression across the stimulation periods (*F*(2.13, 14.93) = 50.32, *p* < 0.0001). Immediately after injection, the normalized H_max_/M_max_ ratio decreased to approximately 68% of the baseline value, indicating an early reduction in spinal excitability. Following the fatiguing stimulation, excitability continued to decline, reaching 48% at PF2 and 36% at PF3. By the end of the recovery phase (PF4), the reflex amplitude had fallen further to about 23% of baseline, representing a nearly 77% depression relative to the prefatigue condition (Figure 1B).

In animals treated with Nifedipine, the depression of the H-reflex was markedly stronger and more persistent (*F*(1.37, 9.61) = 18.59, *p* = 0.001). After injection, excitability decreased to approximately 70% of baseline at PF1, then declined sharply to 33% and 29% during PF2 and PF3, respectively (*p* < 0.001 vs. Pre). Only minimal recovery was observed at PF4, where excitability reached about 40% of baseline, still significantly lower than prefatigue levels (*p* < 0.001). The lack of significant differences between PF2, PF3, and PF4 indicates that reflex depression plateaued during the postfatigue phase, with little evidence of recovery even after 45 min (Figure 1C).

In contrast, (−)-Bay K8644 treatment produced a distinctly different response pattern (*F*(1.80, 12.61) = 5.77, *p* = 0.019). Following drug administration, reflex excitability decreased only slightly to 75% of baseline at PF1 and remained stable during PF2 (82%) and PF3 (77%). By the final recording (PF4), excitability exceeded baseline, reaching approximately 129% of the prefatigue value (*p* < 0.05, Figure 1D). This finding suggests that activation of L-type calcium channels not only counteracted fatigue-induced depression but also facilitated a supranormal recovery of spinal excitability during the late post-stimulation phase.

Collectively, these findings demonstrate that fatiguing high-frequency electrical stimulation produced a robust depression of the H-reflex amplitude, and that calcium-channel modulators distinctly altered both the magnitude and recovery dynamics of this response. Whereas Amiloride and Nifedipine attenuated reflex excitability, (−)-Bay K8644 facilitated postfatigue restitution, suggesting differential effects of calcium-channel blockade and activation on spinal motoneuron responsiveness.

**Figure 2:**
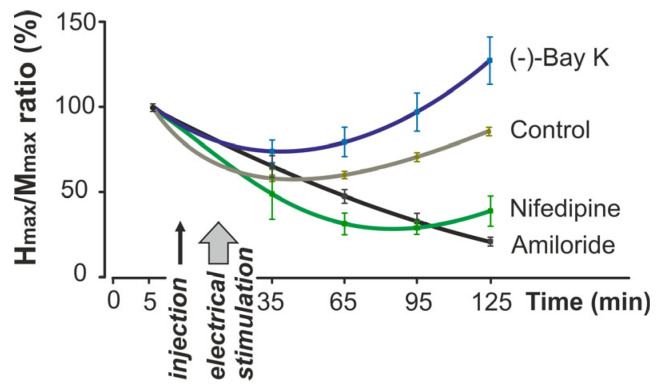
Changes in H_max_/M_max_ ratio during the 125-min experimental protocol. Values are expressed as a percentage of the baseline measurements. Drug injection and electrical stimulation points are indicated on the timeline. The curves represent mean values ± SEM for each experimental group (Control, Amiloride, Nifedipine, and (−)-Bay K8644; *n* = 8 per group).

The time-course analysis (Figure 2) demonstrated distinct group-specific dynamics of the H_max_/M_max_ ratio across the 125-min experiment. Whereas control animals showed partial recovery, Amiloride and Nifedipine produced a progressive decline, and (−)-Bay K8644 elicited a delayed facilitation of reflex excitability above baseline.

**Figure 3:**
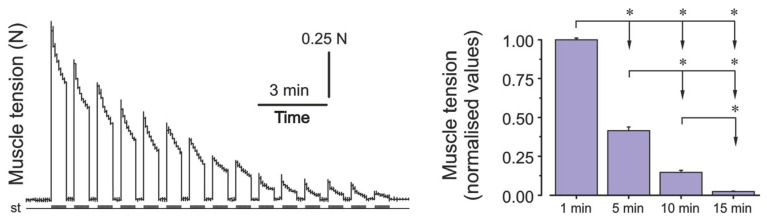
The tensometric analysis of the plantar muscle strength during fatigue development. Representative mechanogram in one of the animals (**left panel**) showing progressive decline in the muscle tension over 15 min of stimulation. Group-averaged normalized muscle force values (mean ± SEM) at four time points (1, 3, 5, and 15 min) in an additional cohort of rats (*n* = 8) are presented in the **right panel**. Asterisks indicate significant reductions relative to baseline (*p* < 0.05). St—stimulation marks; N—muscle tension in Newtons.

To validate the development of skeletal muscle fatigue induced by high-frequency electrical stimulation, tensometric analysis of plantar muscle force was conducted in an additional group of animals. Muscle force was recorded continuously throughout the stimulation protocol. After normalization and averaging, representative values were extracted and presented at four time points: at the onset of stimulation and at 5-min intervals thereafter. The data, shown in Figure 3, demonstrate a clear progression of fatigue across the stimulation period.

## 3. Discussion

This study examined the effects of two calcium channel blockers, Amiloride and Nifedipine, and one calcium channel activator, (−)-Bay K8644, on the modulation of spinal excitability during fatigue of the rat plantar muscle, as assessed by changes in the H-reflex amplitude and the H_max_/M_max_ ratio. In untreated animals, high-frequency electrical stimulation of the sciatic nerve induced a marked reduction in the H-reflex, which was consistent with fatigue-related depression of motoneuron excitability, followed by gradual recovery. Pharmacological modulation of calcium channels substantially altered this pattern. Amiloride and Nifedipine both potentiated the decline in the H-reflex, although through potentially distinct mechanisms, while (−)-Bay K8644 prevented immediate postfatigue depression and ultimately increased the H_max_/M_max_ ratio above baseline. Calcium channels can be broadly divided into low-threshold (T-type) and high-threshold (L-type and others) subtypes, which differ in activation voltage and physiological function. Amiloride predominantly blocks low-threshold channels [22], whereas Nifedipine and (−)-Bay K8644 act on high-threshold L-type channels [23,24,25], explaining their opposite effects on motoneuron excitability and fatigue-related H-reflex modulation. These findings highlight that modulation of calcium fluxes, either at the muscle or neural level, significantly affects spinal reflex responses during fatigue and point to both peripheral and central contributions to these effects.

Fatigue emerges from the interaction of peripheral and central processes. Peripheral fatigue involves disturbances in ionic homeostasis, reduced Ca^2+^ release from the sarcoplasmic reticulum, impaired myofilament sensitivity, and depletion of metabolic substrates [7,26,27]. Central fatigue reflects decreased excitatory drive from supraspinal centers or reduced motoneuron responsiveness [28,29,30]. The H-reflex, widely used to probe motoneuron pool excitability [18,20,21], is sensitive to both muscle-derived afferent feedback and descending modulation. Thus, drug-induced changes in H-reflex amplitude during fatigue reflect an interplay of peripheral excitation–contraction mechanisms and spinal network modulation.

Amiloride induced a sustained decrease in the H_max_/M_max_ ratio across fatigue stages. Known primarily as an epithelial sodium channel blocker, Amiloride also inhibits low-threshold T-type Ca^2+^ channels [22]. In skeletal muscle, blockage of Na^+^–Ca^2+^ exchange reduces Ca^2+^ uptake and thereby accelerates fatigue by impairing excitation–contraction coupling. Amiloride effectively blocks low-threshold Ca^2+^ channels in isolated chicken dorsal medullary ganglia and mouse neuroblastoma cells, but has little effect on high-threshold L-type Ca^2+^ channels [22]. Zavecz et al. [31] demonstrated that inhibition of Na^+^–Ca^2+^ exchange by Amiloride in diaphragm muscle attenuated contractile responses and enhanced fatigue development, likely due to impaired Ca^2+^ uptake. In our experiments, this mechanism likely contributed to reduced muscle force and diminished afferent input to the spinal cord. Although Amiloride is capable of penetrating the blood–brain barrier (BBB) [32], suggesting that partial central involvement cannot be excluded, the predominant effects in our model appear to be peripheral.

Nifedipine produced the strongest suppression of reflex excitability, reducing the H_max_/M_max_ ratio by up to 81%. Nifedipine blocks L-type Ca^2+^ channels, which act as voltage sensors in excitation–contraction coupling [23,33,34]. Although direct Ca^2+^ influx through DHPRs is not essential for single twitches [35], these channels mechanically activate ryanodine receptors to initiate Ca^2+^ release [27]. Their blockage, therefore, compromises contraction and enhances fatigue, which is consistent with our results. Beyond muscle, dihydropyridines can penetrate the BBB [36] and can also suppress neuronal L-type currents involved in dendritic excitability in hippocampal sections in vitro [37]. Human studies have confirmed that Nifedipine reduces exercise tolerance and increases lactate accumulation [38]. Chick and co-authors reported that Nifedipine administration limits peak performance and increases the plasma lactic acid concentration in healthy subjects during veloergometry [38]. This was explained by the fact that Nifedipine reduces blood flow to skeletal muscles, diverting it toward tissues not involved in movement. Moreover, Nifedipine increases catecholamine levels, thereby increasing lactic acid production. Nifedipine has also been shown to reduce skeletal muscle contractility by selectively impairing fatigue-resistant fibers [38]. Thus, its effect on the H-reflex likely reflects both peripheral impairment of contractility and central suppression of motoneuron excitability.

(−)-Bay K8644 had the opposite effect, preventing immediate reflex depression and ultimately increasing the H_max_/M_max_ ratio above baseline. As an L-type Ca^2+^ channel agonist, (−)-Bay K8644 increases Ca^2+^ influx during repetitive activity, facilitating Ca^2+^ entry not only in cardiac and smooth muscle but also in skeletal fibers [16,24,39]. While these channels contribute little to single twitches [40,41], their role becomes critical during sustained mechanical responses such as contractures or tetanic contractions, where extracellular Ca^2+^ influx supports force maintenance [42,43]. Accordingly, Bay K8644 may exert a positive inotropic effect on skeletal muscle fibers [44]. Moreover, its actions are concentration-dependent: at low micromolar levels, it can even interfere with repetitive action potential generation [44,45], suggesting possible interactions with sodium conductance. Importantly, Bay K8644 can also cross the blood–brain barrier [46], where it potentiates neuronal L-type Ca^2+^ currents and may indirectly modulate spinal excitability via monoaminergic pathways [25,47,48,49]. This dual peripheral and central action likely explains the delayed facilitation of the H-reflex observed in later stages of fatigue.

Taken together, the three modulators illustrate how calcium channels regulate both peripheral muscle function and central reflex pathways. Amiloride and Nifedipine reduced Ca^2+^ availability, thereby worsening fatigue and suppressing excitability, whereas Bay K8644 enhanced Ca^2+^ entry, preserving function. While peripheral mechanisms predominate, the BBB penetration of dihydropyridines and their potential effects on central neurotransmission indicate that spinal circuits are also likely involved.

*Limitations*. Several limitations should be acknowledged. First, the sample size was modest (*n* = 8 per group), although the observed effect sizes were substantial across animals. Second, all the experiments were conducted under ketamine/xylazine anesthesia, which may influence neuronal excitability and reflex responsiveness. While this anesthetic combination is commonly used in neurophysiological studies, its potential effects on spinal circuitry and possible interactions with the tested compounds should be considered when interpreting results related to central mechanisms. Third, although some of the tested compounds are known to cross the blood–brain barrier, tissue and brain concentrations were not measured in this study. Consequently, any interpretation involving central mechanisms remains speculative. Finally, this study focused on muscle fatigue induced by high-frequency electrical stimulation, which may engage specific calcium channel subtypes differently from other fatigue paradigms. Future research should determine whether similar modulatory patterns occur under low-frequency or intermittent stimulation protocols.

From a translational standpoint, our findings highlight both the potential and the limitations of calcium channel modulators. While (−)-Bay K8644 facilitated H-reflex recovery in rats, it is important to note that dihydropyridine agonists can exhibit toxicity at high concentrations, particularly through excessive Ca^2+^ entry in cardiac and neuronal tissues. However, evidence suggests that at lower concentrations, such compounds may exert beneficial effects without overt toxicity. Nevertheless, the pharmacological profile of (−)-Bay K8644 is complex, and its lack of clinical use reflects concerns about safety and systemic effects. Future research should therefore aim to identify or develop agents with similar modulatory effects on Ca^2+^ handling but with improved safety margins, ensuring their potential applicability in clinical or sports-related contexts.

## 4. Materials and Methods

### 4.1. Procedure and Experimental Animals

Male and female Wistar rats, weighing 300–330 g, were used in the study. The animals were taken from the vivarium of the Bogomoletz Institute of Physiology (Kyiv). The rats were housed in Plexiglas cages, and kept in a room with air filtration and temperature control (21 ± 1 °C) under 12 h light/12 h dark conditions. The animals received a standard pellet diet and water ad libitum. The use of the animals was approved by the Committee for Biomedical Ethics of the Institute (#2/22, 26 February 2020) and performed in accordance with the European Union Directive of 22 September 2010 (2010/63/EU) for the protection of animals used for scientific purposes. All procedures complied with the ARRIVE guidelines.

The animals were randomly assigned to experimental groups using a computer-generated randomization sequence. The six groups of animals were used in the study: (1) control animals without injections (*n* = 8); (2) vehicle-injected animals: rats receiving an intraperitoneal (i.p.) injection of saline solution, 0.3 mL (*n* = 8); (3) animals receiving an i.p. injection of the calcium channel blocker Amiloride, 1 mg/kg (*n* = 8); (4) animals receiving an i.p. injection of the calcium channel blocker Nifedipine, 5 mg/kg (*n* = 8); (5) animals receiving an i.p. injection of the calcium channel activator (−)-Bay K8644, 0.5 mg/kg (*n* = 8); and (6) animals for tensometric assessment of fatigue (*n* = 8). At this dose, (−)-Bay K8644 did not induce spasticity in rats. Doses were selected to fall within published in vivo ranges that modulate Ca^2+^ handling while minimizing off-target toxicity [50,51,52]. Previous studies have shown that Bay K8644 reaches maximal systemic effects within 30–40 min after i.p. injection [25], whereas Amiloride and Nifedipine display rapid onset following intravenous administration [53,54]. Therefore, the interval between drug administration and the end of electrical stimulation (35 min) was chosen to ensure sufficient systemic exposure for all agents.

Amiloride, Nifedipine, and (−)-Bay K8644 of the highest purity were purchased from Sigma-Aldrich Inc. (St. Louis, MO, USA).

### 4.2. Surgical Preparation and Electrical Stimulation Protocols

Before the experiments were performed, the rats were anesthetized with ketamine (100 mg/kg, Pfizer, New York, NY, USA) and xylazine hydrochloride (10 mg/kg, Interchemie, The Netherlands). The sciatic nerve (*n. ischiadicus*) was exposed and carefully separated from the surrounding tissue. The animals were placed on a platform with rigid fixation of the head, pelvis, and limbs. The sciatic nerve was mounted on two chlorided silver electrodes positioned ~5 mm apart. Hindlimb muscles and nerves were covered with paraffin oil in a skin flap pool to prevent drying.

Muscle fatigue was induced by direct electrical stimulation of the plantar muscle using two chlorided silver electrodes (0.15 mm in diameter) inserted 2–3 mm into the muscle via injection needles. The threshold current was defined as the minimal intensity eliciting a visible contraction, corresponding to the activation of low-threshold Ia afferents. Stimulation was delivered with 0.2 ms rectangular pulses at a 1.5× threshold intensity, applied at 50 s^−1^ [4] for 15 min (40 s stimulation, 20 s rest per minute) [55,56].

**Figure 4:**
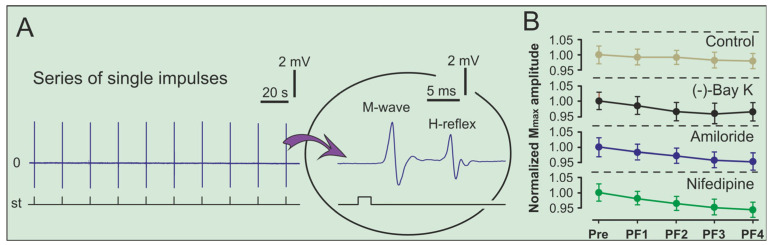
Combined representation of the electrical stimulation protocol and the M-wave stability analysis. (**A**) **Left panel**: Upper row—representative series of the H-reflex and M-wave recordings evoked by ten single electrical impulses of one of the animals in Group 4 (control series); Lower row—corresponding stimulation trace showing the timing of each impulse (st—a series of subsequent electrical stimuli). **Right panel**: Expanded view of the final impulse, illustrating the temporal separation of the H-reflex and M-wave components. (**B**) Significant decrease of the M-wave amplitude (mean ± SEM), normalized to prefatigue baseline, shown separately for each experimental group (*n* = 8, for all groups).

For H-reflex and M-wave recordings, a series of 10 rectangular pulses (0.2 ms duration, 20 s interstimulus interval) was applied to the sciatic nerve (Figure 4A). Responses were recorded from the plantar muscle using two chlorided silver electrodes (0.15 mm in diameter) placed 4.0–4.5 mm apart. Throughout surgery and experimentation, heart rate, ECG, and body temperature were monitored, and temperature was maintained by an infrared lamp.

To ensure that no data drift occurred during the experiment, the maximum amplitude of the M-wave (M_max_) was normalized to prefatigue values and analyzed across time points. Although a slight deviation (~5%) in M_max_ amplitude was observed during prolonged stimulation, this variation remained within physiologically acceptable limits and did not reach statistical significance. Given the substantially larger changes in H_max_ amplitude, the observed modulation of the H_max_/M_max_ ratio reflects true alterations in spinal cord excitability rather than variability in M-wave stability (Figure 4B).

**Figure 5:**
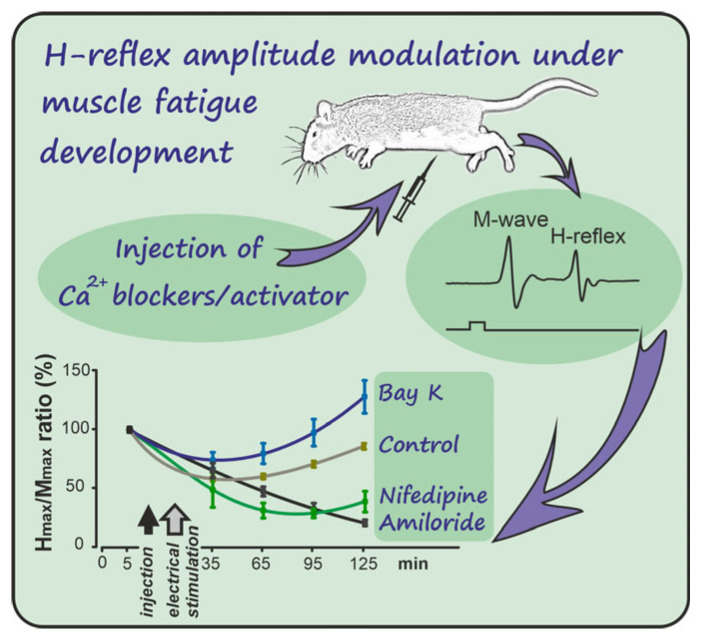
Schematic representation of the study. The calcium channel blocker Amiloride, Nifedipine, and calcium channel activator (−)-Bay K8644 modulate the *m. plantaris* H-reflex amplitude under fatigue development induced by high-frequency electrical stimulation. Bay K markedly increased the H-reflex amplitude of the studied muscle, when the H-reflex technique was used.

The maximum H-reflex amplitude and the M-wave were recorded before (one time) and after (four times) animal treatment. Injections were administered immediately after the first (Pre) recording of the H-reflex amplitude. Twenty minutes after injection [14,15,16], the plantaris muscle was directly stimulated for 15 min to induce fatigue. The second H-reflex recording (postfatigue 1, PF1) was performed immediately after this stimulation, and subsequent recordings were obtained every 30 min (postfatigue 2, PF2; postfatigue 3, PF3; postfatigue 4, PF4). The stimulation pattern was designed such that after the first (control) series of stimulation the H-reflex amplitude was restored by at least 60% of the control data. The general experimental design is shown in Figure 5.

The development of muscle fatigue was estimated by decreases in the H-reflex amplitude and H_max_/M_max_ ratio [26,28]. Indirect signs of muscle fatigue development, such as the absence of visual muscle contraction during electrical stimulation, and the absence of registered signals after a rest period, were also taken into account. To confirm the presence of muscle fatigue induced by the stimulation protocol, tensometric analysis of plantar muscle force was performed in a separate group of animals. The muscle was carefully separated from surrounding tissues and connected to a calibrated force transducer. The plantar muscle was stimulated using the same high-frequency protocol applied in the main experiment, and the resulting tension was recorded continuously. Force values were normalized and analyzed at four time points (1, 3, 5, and 15 min) to assess fatigue progression (Figure 3).

Signals were sampled via a DAC-ADC system (CED Power 1401, Cambridge Electronic Design, Cambridge, UK). DAC output triggered isolated stimulators (model DS2A, Digitimer, Welwyn Garden City, UK) for nerve stimulation. Input signals were amplified (Brownlee model 440, Santa Clara, CA, USA), digitized at 10 kHz, and recorded with Spike2 v.9 software (CED, Cannock, UK).

Following the experiment, the animals were euthanized via Nembutal overdose.

### 4.3. Data Analysis

For each stimulation series, the maximum H-reflex amplitude was averaged across 10 responses, with the first series set as a control. The maximum M-wave amplitudes were also averaged across the 10 stimulations. The H_max_/M_max_ ratios were normalized to those of the control series and are expressed as the mean ± standard error of the mean (SEM). Data normality was assessed using the Shapiro–Wilk test. Differences across the stimulation periods (Pre, PF1, PF2, PF3, PF4) and between the experimental groups were analyzed using a two-way repeated-measures ANOVA, with Time as a within-subjects factor and the Group as a between-subjects factor. When significant effects were found, Bonferroni-adjusted post hoc tests were applied for pairwise comparisons (*p* < 0.05). Additionally, the one-way repeated-measures ANOVAs were performed within each group to assess time-dependent changes. Assumptions of sphericity were evaluated using Mauchly’s test. When violated, the Greenhouse-Geisser correction was applied. Effect sizes were reported as partial eta squared (η^2^_p_), with 95% confidence intervals. Statistical analyses were performed in Origin 8.5 (OriginLab Corporation, Northampton, MA, USA).

The sample size of eight animals per group was chosen on the basis of preliminary data showing large differences in H_max_/M_max_ ratios between treatment conditions (40–100% of baseline values). For effects of this magnitude (Cohen’s d ≈ 1.0–2.0), a group size of eight yields statistical power > 80% at α = 0.05 in repeated-measures designs.

## 5. Conclusions

In conclusion, our findings indicate that pharmacological modulation of calcium channels can markedly influence H-reflex behavior during muscle fatigue development in rats. Amiloride and nifedipine tended to enhance reflex depression, whereas (−)-Bay K8644 attenuated fatigue-induced suppression, suggesting differential effects depending on the targeted calcium channel subtype.

These outcomes likely involve both peripheral alterations in excitation–contraction coupling and potential central contributions, although the latter remain speculative due to the absence of direct measurements of tissue or brain concentrations.

Overall, the results highlight a close interplay between calcium regulation, muscle performance, and spinal reflex pathways. Given the limited sample size and indirect evidence of the underlying mechanisms, these findings should be regarded as hypothesis-generating and warrant further investigation into how calcium channel modulation may influence fatigue resistance and neuromuscular function.

## Data Availability

The data presented in this study are available on request from the corresponding author.

## Abbreviations

The following abbreviations are used in this manuscript:

ANOVA	Analysis of variance
BBB	Blood–brain barrier
DAC-ADC	Digital-to-Analog Converter–Analog-to-Digital Converter
DHPR	Dihydropyridine receptor Ca^2+^ channel
Pre	Prefatigue recordings of H-reflex amplitudes
PF1–PF4	Postfatigue recordings 1–4 of H-reflex amplitudes in different periods of time
RyR	Ryanodine receptor
SEM	Standard error of mean
SR	Sarcoplasmic reticulum
